# One minute of circulatory arrest for acute type A aortic dissection --------- a simple operation for acute type A aortic dissection (AAAD)

**DOI:** 10.1186/s13019-020-01370-1

**Published:** 2020-11-12

**Authors:** Detian Jiang, Yufeng Huo, Yimin Liu, Yan Wang, Jinfeng Zhou, Xiangfei Sun, Fen Zhao, Yonghai Du, Songxiong He, Chao Liu, Wenyu Sun

**Affiliations:** 1 Department of Cardiovascular Surgery, Qilu Hospital of Shandong University, Qingdao, 266000 China; 2 Department of Cardiovascular Surgery, Shandong Provincial Hospital Affiliated to Shandong University, Jinan, China

**Keywords:** Circulatory arrest, Acute type a aortic dissection, Operation, Sun’s procedure, One minute

## Abstract

**Background:**

Sun’s procedure is currently recognized as the standard procedure for acute type A aortic dissection (AAAD). But the operation istoo difficult for beginners. We hope to reduce the difficulty and complications of this operation.

**Methods:**

The aortic arch was immediately cross-clamped after the stented graft was inserted into the distal aorta. Thereafter, the lower-body perfusion was restored. Then, anastomosis was performed between the proximal stent graft and the distal 4-branched Dacron graft. The other arteries were anastomosed to the arched branch of the 4-branched graft.

**Results:**

The cardiopulmonary bypass (CPB) time was (207 ± 52) min, and the aortic cross-clamp time was (114 ± 39) min. The circulatory arrest time was (38 ± 16) sec. One patient (4%) died. The incidence of complications was stroke (4%), renal dysfunction requiring dialysis (4%), prolonged intubation(12%).

**Conclusions:**

The time of circulatory arrest in this operation is less than 1 min, which can avoid the complications caused by DHCA and decrease risk of bleeding and complexity by shifting anastomosis more proximally. The effect of our operation is similar to and even better than that of Sun’s procedure. It does not even require relatively advanced skill, much experience and excellent psychological quality, especially suitable for beginners.

## Background

Acute type A aortic dissection (AAAD) remains one of the most challenging diseases for cardiothoracic surgeons. In spite of technical improvements, the perioperative mortality rate is stillvery high. In the early stage of AAAD, the mortality rate increases by approximately 1% per hour [1–3]. Surgery is the most effective treatment. At present, the most common surgical methods for the treatment of AAAD are Sun’s procedure and hybrid operations [4]. Sun’s procedure is widely used because of its advanced techniques and good results,while it requires deep hypothermic circulatory arrest (DHCA). Hybrid surgeries can avoid DHCA, but they have more frequent long-term complications. We hope to reduce the difficulty of this operation on the basis of ensuringsurgical outcome. Our center tried a variety of surgical methods for the treatment of AAAD. At last,we coined one surgery which is based on Sun’s procedure and the idea of debranching. The time of circulatory arrest needed during this surgery is only 1 min.

Herein, we give a report incorporating 25 patients who were treated with this operation between January 2017 and December 2018, retrospectively.

### Patients and methods

The retrospective study included 25 patients who underwent surgical treatment in Qilu Hospital of Shandong University between January 2017 and December 2018. All patients were diagnosed with AAAD by aortic computed tomography angiography (CTA)before the operation.

### Operative indications

1. type A dissections with the primary entry locating in the arch and descending aorta
2. type A dissections severely involving the archvessels
3. type A dissections with extensive intimal intussusception
4. Marfan syndrome complicated with type Adissection

This study was approved by the Ethics Committee of Qilu Hospital of Shandong University. Their clinical data are summarized in Table 1.

**Table 1:** Clinical Data

	Number	Mean ± SD
Sex M:F	20:5	
Age (years)		44 ± 11
Hypertension	21(84%)	
Marfan syndrome	1(4%)	
Moderate to severe aortic regurgitation	5(20%)	
Cardiac tamponade	1(4%)	
Coronary ischemia	2(8%)	
Renal insufficiency	3(12%)	
Smoking, past or current	17 (68%)	
Chest or abdominal pain	25(100%)	
Neurological symptoms	4(16%)	
Pulmonary disease	2(8%)	

### Surgical materials

**Fig. 1:**
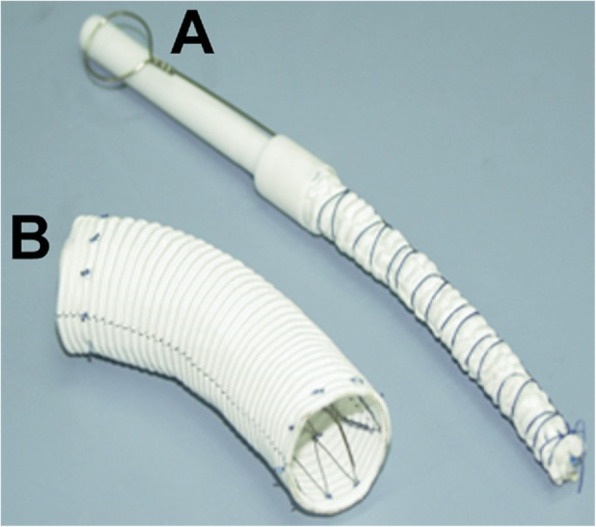
The stented elephant trunk, Cronus®, before (**a**) and after (**b**) implantation [6].

The materials were as same as which were used in Sun’s procedure. The stented graft is 150 mm long and 28 to 32 mm in diameter. It consists of a Gianturco-type self-expandable metallic stent (Microport Medical Corp) and a high-porosity woven Dacron graft (Intervascular OLP, Intervascular Inc). At each end, there is a10-mm-long stent-free sewing edge which could be clamped without affecting the overall performance of the stent [5, 6]. 120 mm stented grafts were used in some women who were especially short.. It was produced by Shanghai Minimally Invasive Company. (Fig. 1).

**Fig. 2:**
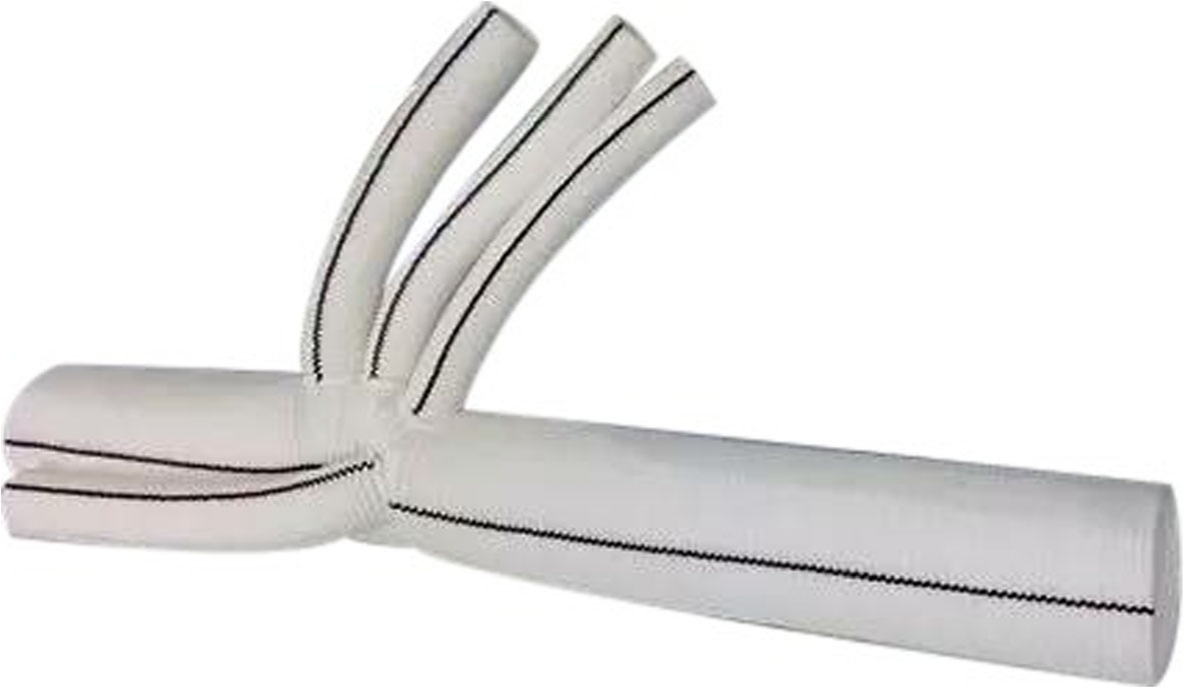
The 4-branched vascular graft (10 × 8 × 8 × 10 mm)

The 4-branched vascular graft (28 to 32 mm in diameter, 10 × 8 × 8 × 10 mm) was a product of Maquet Company [5, 6]. (Fig. 2).

During perioperative period,we treated the patients with oxygen,analgesia, maintain arterial pressure and heart rate. The operation was performed under general endotracheal anesthesia with continuous transesophageal echocardiography, cerebral oxygen saturation (rSO2), and arterial pressure monitoring.

### Operative technique

**Fig. 3:**
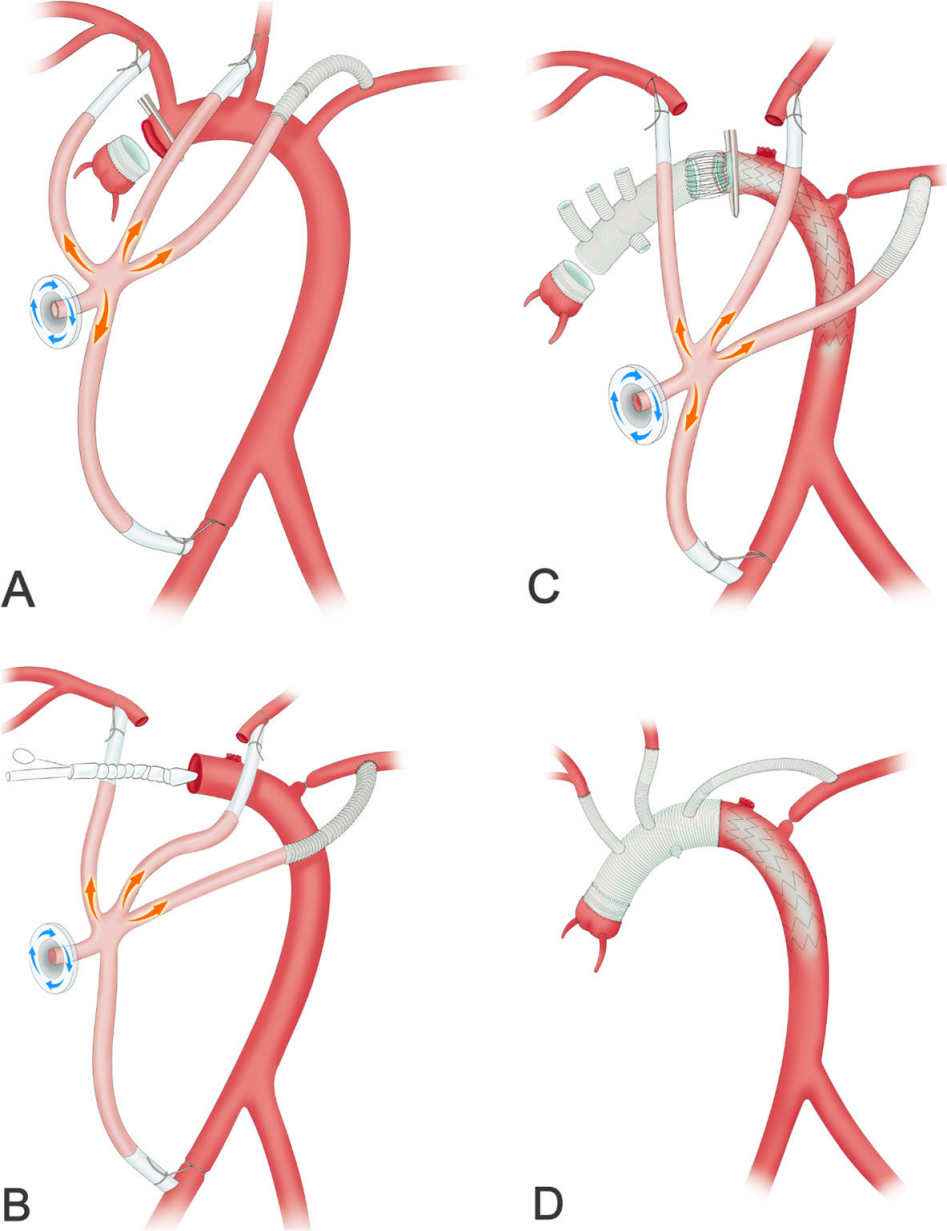
The surgical procedure. **a** The aortic root was repaired after CPB was established. **b** The intraoperative stent was inserted into the distal aorta. **c** The anastomosis was performed between the proximal stent graft and the distal 4-branched graft after the aortic arch was cross-clamped. **d** The other arteries were anastomosed to the arched branch of the 4-branched graft

After sternotomy, the patient was heparinized. A 8 mm (15 cm length) graft was end-to-side anastomosed to the left subclavian artery and the other end of this graft was tunneled via the second intercostal space into the mediastinum connected to the arterial tubes of the the cardiopulmonary bypass (CPB) machine. The femoral artery, innominate artery, left common carotid artery were cannulated with arterial catheters, which were connected to the arterial tubes of the CPB machine. Cooling was started after cannulation of the superior and inferior vena cava. The ascending aorta was cross-clamped at 30 °C and the cardioplegic solution was usually perfused through a coronary sinus cannulation to arrest the heart. After the cardiac arrest, the aortic valve repair or replacement was performed if significant aortic valve insufficiency was identified. The ascending aorta was also replaced by the graft,which was anastomosed to the aortic sinotubular junction or the artificial valve ring. (Fig. 3a).

The innominate artery and the left common carotid artery were transected. Thereafter, the left subclavian artery was ligated proximally. The cross-clamp was moved to the distal aortic arch (between the innominate artery and the left common carotid artery), and the aortic arch was trimmed. Hypothermic circulatory arrest was started after removing the clamp. The intraoperative stent was inserted into the true lumen of the distal aorta in a bound, compressed state after the distal aorta was transected between the origin of the innominate artery and the left common carotid artery. Open the stent to support the inner wall of the aorta. (Fig. 3b).

The aortic arch was immediately cross-clamped after de-airing. Thereafter, the lower-body perfusion was restored. The self-aortic arch and the intraoperative stent’s graft section were trimmed, and the sandwich treatment was performed. Then, anastomosis was performed between the proximal stent graft and the distal 4-branched Dacron graft. (Fig. 3c).

The previously placed graft on the root was retracted,stretched and measured to the site of proximal 4-branched Dacron graft. Graft-to-graft anastomoses were performed. Finally, the innominate artery, the left common carotid artery and the 8 mm graft of left subclavian artery were anastomosed to the arched branch of the 4-branched graft in proper sequence. (Fig. 3d).

The similarities and differences between our operation and Sun’s procedure are summarized in Table 2.

**Table 2:** The similarities and differences between our operation and Sun’s procedure

	Sun’s procedure	Our operation
Similarities	operative Indications surgical materials anesthesia cannulation of the vena cava repair of aortic root	
Differences		
Incision	median sternotomy right infra-clavicular	median sternotomy left infra-clavicular inferior inguinal ligament
Artery perfusion	right axillary artery left common carotid artery	femoral artery, innominate artery, left common carotid artery left subclavian artery
Left subclavian artery	transected, end-to-end anastomosed to the arched branch of the 4-branched graft	ligated, A 8 mm graft was end-to-side anastomosed to the left subclavian artery and the other end of this graft was tunneled via the second intercostal space into the ediastinum connected to the arched branch of the 4-branched graft
Cerebral perfusion	selective	bilateral
Temperature of circulatory arrest	deep hypothermia	30 °C
Time of circulatory arrest	more than 20 min	less than 1 min
Lower-body perfusion when anastomosis was performed between the proximal stent graft and the distal 4-branched graft	No	the aortic arch and stent graft were cross-clamped, lower-body perfusion was restored by the femoral artery
Sit of anastomosis between the proximal stent graft and the distal 4-branched graft	descending aorta distally to the origin of the native left subclavian artery	aortic arch between the innominate artery and the left common carotid artery

## Results

We compared our results with Sun’s procedure completed at two of the best cardiac centers in China. In addition, a systematic review and meta-analysis was conducted. The results showed that the follow-up data in our operation were comparable or superior to the data in patients with Sun’s procedure. The circulatory arrest time、lowest nasopharyngeal temperature and blood loss in our operation were less than that in Sun’s procedure. And there were lower operative mortality rate and fewer complications in our operation.

Operative Data and are reported in Table 3.

**Table 3:** Operative Data

	Our operation		Sun’s procedure	
	Number	Mean SD	Number	Mean SD
Bentall procedure	1 (4%)		30% [7] 29% [8]	
Reconstruction of the sinus of Valsalva	5 (20%)		17% [7] 33% [8]	
Coronary artery bypass graft	1 (4%)		9% [7]	
CPB (min)		207 ± 52		201 ± 51 [7] 196 ± 63 [8]
Aortic cross-clamp time (min)		114 ± 39		111 ± 31 [7] 100 ± 29 [8]
Circulatory arrest (sec)		38 ± 16		1440 ± 480 [7] 1390 ± 487 [8]
Surgery duration (min)		463 ± 136		408 ± 125 [8]
Lowest nasopharyngeal temperature(°C)		30		25 [7] 20 [8]
Blood loss during operation (mL)		841 ± 85		947 ± 773 [8]
Blood product use				
Red cell (U)		6.7 ± 6.3		6.8 ± 8.8 [8]
Fresh Frozen Plasma (mL)		845 ± 692		873 ± 1024 [8]
Platelet (U)		2.3 ± 1.9		2.4 ± 2.6 [8]

Postoperative complications are reported in Table 4.

**Table 4:** Postoperative Complications

Postoperative complications	Our operation	Sun’s procedure
In-hospital death	1 (4%)	7.8% [7] 10.7% [8] 8.6(7.0–10.2)% [9]
Stroke	1 (4%)	4.4% [8] 3.7 (2.1–5.7) % [9]
Renal dysfunction requiring dialysis	1 (4%)	4.3% [7] 9.6% [8]
Prolonged intubation (including Tracheostomy)	3 (12%)	17.1 (10.9–24.4) % [9]
Recurrent nerve palsy	0	1% [7]
Paraplegia	0	1.8% [7] 4.4% [8] 1.95 (1.04–3.12) % [9]
Hepatic insufficiency	0	31.4% [8]
Reexploration for bleeding	0	2.5% [7] 3.7% [8]

The follow-up for patients were from 18 months to 42 months. The interval of CTA was 6 months after the operation.

## Discussion

Sun’s procedure is currently recognized as the standard procedure for AAAD. The incidence of residual distal false lumen patency from Sun’s procedure is less than 5%, and the reoperation rate is less than 10% [10–12].

DHCA is indispensable for Sun’s procedure. But DHCA may cause abdominal organ dysfunction such as ischemia-reperfusion injury, coagulation dysfunction, nervous system dysfunction and kidney dysfunction. Besides, the incidence of these dysfunction is positively correlated with the duration of DHCA [13–16].

Hybrid surgery can reduce the difficulty of the operation and recent complications by avoiding DHCA, but it also leads to a relatively high incidence of late complications [17, 18], due to its lack of one important step of Sun’s procedure that the expandable stent graft can be firmly fixed to the distal 4-branched prosthetic graft using the suture line. One study reported that the incidence of late complications was up to 48%, including delayed type I endoleak, stent migration, stent fracture and so on. Furthermore, 10% of the patients underwent late open surgery [19, 20].

In order to solve these problems, we coined a new surgery which is a combination of Sun’s procedure and hybrid surgery. In this surgery, we proposed that we could use 1 min of circulatory arrest to place the expandable stent graft into the aorta. This amount of time is so short that there is no need for deep hypothermia.

The time that DHCA is safe is 30–40 min; the shorter the time is, the better [21, 22]. If surgeons can not complete high-quality complex surgery as soon as possible during safe operational time window, patients may be left with serious complications. So they need relatively advanced skill, much experience and excellent psychological quality.

On the contrary, a surgeon does not need to worry about the time of circulatory arrest in our surgery. Thus, it apparently reduces the psychological pressure on the surgeon, which is conducive to a better effect.

When the distal aortic arch is anastomosed, bilateral anterograde cerebral perfusion and retrograde femoral artery perfusion are adopted. There is theoretical possibility of retrograde tear of the dissection pseudolumen during retrograde femoral artery perfusion. However, some research shows that this approach is safe and reliable [23].

In Sun’s procedure, it is difficult to handle the root of the left subclavian artery, and the recurrent laryngeal nerve can be easily injured. End-to-side anastomosis was performed between the graft and left subclavian artery via a left infraclavicular incision in our surgical procedure. During the operation, the left vertebral artery could be perfused continuously through the graft, which is helpful for protecting the brain and spinal cord. The distal anastomosis was located between the innominate artery and the left carotid artery, which was relatively simple and easy to be exposed. Then, sutures were placed, and the hemostasis was maintained. The risk of bleeding and recurrent laryngeal nerve injury was reduced. Since the three branches of the aortic arch were perfused continuously, they were anastomosed after the reconstruction of the aortic arch so that the length and location of the branches were easier to be adjusted, which further reduced the difficulty of the operation.

In the study, one patient experienced aggravation of renal damage from preoperative renal insufficiency to postoperative renal failure After treatment with continuous renal replacement therapy (CRRT), his renal function returned to be normal. No paraplegia, liver failure or other abdominal organ ischemia complications occurred in this study. The adoption of continuous perfusion of the subclavian artery and less than 1 min circulatory arrest reduced the risk of ischemia and ischemia reperfusion injury. Therefore, this approach is beneficial for the protection of the spinal cord and abdominal organs and allows for the possibility of reducing the incidence of related complications.

In addition, another advantage of our operation is that the location of anastomosis on the aorta is altered from the left subclavian artery to the innominate artery, which decreases the risk of bleeding and complexity by shifting anastomosis more proximally.

It is also one of the aprroaches to prevent DHCA that coda balloon or Foley’s catheter is used to block descending thoracic aorta and then lower body can be perfused by femoral artery, which can also protect abdominal organs,which can also protect abdominal organs [24–26]. However, compared with our operation, there are some disadvantages, such as blood leakage around the balloon and unclear vision when the distal end is sutured. What’s more, the anastomosis is still too far for the surgeon, which cannot reduce the difficulty of operation.

### Limitations

The number of locations for anastomoses was more in this procedure than that in Sun’s procedure; thus, the overall operation time of this operation was appropriately longer than that of Sun’s procedure.

## Conclusions

The time of circulatory arrest in this operation is less than 1 min, which can avoid the complications caused by DHCA and decrease risk of bleeding and complexity by shifting anastomosis more proximally..The effect of our operation is similar to and even better than that of Sun’s procedure. The most important is that this operation is much easier than Sun’s procedure. To some extent, this procedure can be regarded as a simple version of Sun’s procedure. It does not even require relatively advanced skill, much experience and excellent psychological quality, especially suitable for beginners.

## Data Availability

The datasets during and/or analysed during the current study available from the corresponding author on reasonable request.

## Abbreviations

AAAD: Acute type A aortic dissection
CPB: Cardiopulmonary bypass
CTA: Computed tomography angiography
DHCA: Deep hypothermic circulatory arrest
CRRT: Continuous renal replacement therapy
